# Editorial: How social and personal resources support teaching and learning effectiveness

**DOI:** 10.3389/fpsyg.2023.1137501

**Published:** 2023-01-25

**Authors:** Ramona Paloș, Delia Vîrgă

**Affiliations:** Department of Psychology, West University of Timiṣoara, Timiṣoara, Romania

**Keywords:** social and personal resources, teaching, learning, secondary and higher education, academic performance

This editorial summarizes the contributions to the Frontiers Research Topic “*How social and personal resources support teaching and learning effectiveness*,” established under the Educational Psychology section and appearing under the Frontiers in Psychology journals.

This Special Issue explored how personal and social resources can impact teaching and learning and how teachers and students value them in dealing with different challenging tasks in the educational environment. Thus, nine articles were accepted for publication. Empirical studies and systematic reviews were considered to answer the following questions: *Are personal or social resources more significant for the effectiveness of teaching and learning, or does their impact depend on the context? How can we develop these resources? How do these resources shape academic performance, job satisfaction, and, finally, the wellbeing of teachers and students*?

Thus, Hoferichter et al. explored the interaction of two social resources (i.e., teachers and peer support) with stress and academic achievement, both on individual and class levels. They worked with 733 7th and 8th-grade students, and the results revealed that teachers perceived support is associated with students' ability to cope with stressful situations and lower levels of helplessness. On the class level, peer support was related to a higher ability to cope and academic achievement. The context effects also show that in classes with higher peer support, students are more likely to benefit in terms of coping ability and achievement. In classes with higher teacher support, students tend to show less coping ability. A specific stressful situation in an academic environment is represented by the exams students must pass. Thus, Schürmann et al. worked with 92 students to investigate whether there are naturally occurring profiles based on the examinee's basic need strength and perceived need for support in real-life oral exams and if these profiles differed in stress responses and achievement. The results revealed two higher-quality (low/high, high/high) and two lower-quality (low/low, high/low) need strength/need support classes. Higher-quality classes that met or exceeded the needs displayed more beneficial stress and emotional response patterns than lower-quality classes. Gain-related emotions mediated achievement in the higher-quality classes. These findings emphasize the necessity to consider learners' emotional states and needs in teachers' didactic efforts because the perceived need for support and satisfaction can shape emotional and physiological stress reactions during the exam. **Martinot et al.** included parental involvement in their research and investigated whether parents, peers and teachers are the best sources of social support for school engagement. Based on 623 middle-school students from a privileged or priority education area, the results showed that the mother provided more support, followed by the father, the teachers, and the peers. Also, each source of social support contributed to school engagement (except for maternal support). Emerging as the best source of support for school engagement, peers and teachers had significant direct effects among students from the priority education area and both direct and indirect effects among students from the advantaged area. Additionally, peer support had a double-edged impact on school engagement among students in the priority education area. Shao and Kang focused on how adolescents' peer relationships are linked to learning engagement through the chain mediating roles of self-efficacy and academic resilience. Results indicated that peer relationship was indirectly but positively associated with learning engagement *via* self-efficacy and academic resilience, respectively, and sequentially. More importantly, the authors found that the direct effect was much lower than the indirect effects, of which self-efficacy was the greatest. This result suggested that adequate interventions should be provided to facilitate adolescents' peer relationships, self-efficacy, and academic resilience, thus promoting their learning engagement and, also, academic success.

The role of student engagement was also investigated by Ma and Wei. They were interested in its mediating role in the relationship between perceived classroom climate and academic performance, according to the motivation process of the study demands-resources (SD-R) model. Thus, working with 307 English-major teacher education students in Guangxi, China, they found that perceived classroom climate (an environmental resource) enhances student engagement (a personal resource), academic performance (a study outcome), and student engagement partially mediates the relationship between the two variables. Because many studies used concepts and measurement tools related to “school climate” as substitutes for school support, Li et al. made a systematic literature review of core Chinese- and English-language journals published in 2000–2021 to analyze school support's concept and measurement tools. Their research shows that school support is mainly approached through two disciplines, namely psychology and pedagogy. The theoretical foundation is provided especially by social support, ecosystem, and school climate theories. Many studies emphasize the values and school climate that contribute to creating a sense of safety in the school and influence the quality of interpersonal relationships that shape the support students receive from teachers. Also, it is helpful to develop and validate school support measurement tools with good psychometrics properties to provide a practical reference for educators worldwide.

Personal resources are also essential in dealing with different challenging tasks in the educational environment. For instance, the last 2 years of experience have shown us the importance of digital skills in managing academic activities. Assante et al. investigated the role of personal resources (i.e., self-direction and universalism) in supporting learning effectiveness related to the digital citizenship development process, namely the critical perspective toward online participation and the Internet. Working with 536 Romanian students in various social sciences domains, their findings illustrated that only higher universalism relates to sustainable digital citizenship, while self-direction has no effect. Also, individual orientation toward information-seeking endorses digital citizenship and a critical perspective toward online participation and the Internet, while cognitive integrity harmed digital citizenship. Moreover, students with higher universalism reported higher learning orientation. Beyond the need to develop digital competencies, the effects of media overuse on wellbeing cannot be neglected. Toma et al. investigated the protective role of hope on students' wellbeing during the pandemic period when all the academic activities moved into the online environment. Three hundred and thirty-three Romanian students were involved in the study. The results showed significant negative associations between attention problems, smartphone addiction, and wellbeing, with dispositional hope as a protective factor. Although smartphone addiction appeared especially harmful to the wellbeing of students with high dispositional hope, they reported greater levels of wellbeing than those with low levels of hope regardless of smartphone addiction.

Personal resources are also essential for teaching efficacy. Thus, Wang et al. explored the internal mechanisms among teachers' assessment literacy, psychological capital, professional identity, and teaching efficacy, working with 351 secondary school teachers in Henan Province, China. Their findings illustrate that teachers' assessment literacy, as a constructive resource, affects teaching efficacy directly. Their psychological capital and professional identity, as energy resources, mediate the relationship between the two variables. According to the COR theory, this study emphasizes that wealth, individual constructive resources, and energy resources facilitate positive gain spirals in key resources.

Thus, these studies revealed how personal and social resources could help teachers to deal with the challenges of the teaching and learning process and how these resources could help them to be efficient in the teaching process and experience wellbeing in the educational environment. Also, the results of these articles offered details related to each of these resources that could help students to self-regulate the learning process, achieve their academic goals, and experience student engagement.

## Author contributions

RP drafted the manuscript. DV critically reviewed the manuscript. All authors contributed to the article and approved the submitted version.

